# Is there stability in the performance of elite half-marathoners?

**DOI:** 10.1016/j.smhs.2022.02.003

**Published:** 2022-03-04

**Authors:** Mabliny Thuany, Beat Knechtle, Pantelis Theodoros Nikolaidis, Thayse Natacha Gomes

**Affiliations:** a Faculty of Sports, University of Porto, Porto, Portugal; b Institute of Primary Care, University of Zurich, Zurich, Switzerland; c School of Health and Caring Sciences, University of West Attica, Athens, Greece; d Department of Physical Education, Federal University of Sergipe, São Cristóvão, Brazil

**Keywords:** Tracking, Endurance, Running, Elite athletes

## Abstract

The purpose of this study was to investigate the performance stability of elite half-marathoners of both sexes. The study was composed of 24 elite athletes (12 female and 12 male), ranked among the Senior World TOP20 half-marathoners, who completed a half-marathon race for at least three consecutive years. Tracking was tested using autocorrelations and Kappa Cohen. Autocorrelation revealed a significant association, but a decrease in correlation among the years in both sexes. The overall weighted kappa showed lower stability in performance for both sexes (*K* ​= ​0.191 and *K* ​= ​−0.245) than for males. These findings suggest that both female and male elite half-marathoners showed low stability in performance during three events. Besides that, athletes with a better performance tended to present the highest performance stability. It is recommended that coaches track the developmental trajectories of the athletes to comprised the changes in performance across the years, as to provide environmental characteristics that can influence performance.

## Abbreviation list

V̇O_2_max: maximum oxygen consumption
mmol^.^L^−1^: Millimole/liter

## Introduction

Since athletic performance is the result of several factors related to both individual and environmental characteristics,^1^ changes in any of these factors can be linked with changes in the long-term performance of high-level athletes. Regarding long-distance running, few studies were conducted to investigate factors associated with long-term performance.^2^^,^^3^ When these studies were performed, most of them investigated injury risks^3^, ^4^, ^5^ or physiological indexes associated with performance.^2^^,^^6^ For example, a classical case study conducted during five years with one female Olympic athlete showed that the studied athlete improved her performance (i.e., time in the 3000 ​m running event), but this improvement was followed by a decrease in V̇O_2_max with an improvement in running economy.^6^

These results suggest that longitudinal data can provide important key points to athlete’s performance. Among human growth studies, the concept of tracking has been largely used.^7^^,^^8^ Based on the idea of stability, change, and predictability, tracking has been related to the trend to maintain a certain level of state and/or behavior in the long-term,^9^ and it allows to estimate the trajectory of subject or group (e.g., staying on a given position) across time.^10^

Most of the studies about tracking investigated aspects related to epidemiology or biological development.^8^^,^^11^^,^^12^ To study performance over time, it is important to guide the long-term training, and also to identify the stability/instability of the athlete’s performance, i.e., if there is high variability in athlete’s performance across the years. Among the available studies using the tracking approach/concept, few were centered in individual sports, such as endurance. However, in the last years, it has been observed an increase in the number of runners and race events across the world, as well as an improvement in the performance among professional athletes.^13^ Thus, the purpose of this study was to investigate the performance stability in elite half-marathoners of both sexes. We hypothesized that athletes of both sexes present stability in the performance across the years, highlighting small variabilities in the performance.

## Material and methods

### *Approach to the problem*

Data were collected from the results section of the *Tilastopaja* website^14^ during November 2020, and information referred to available results for the world’s best half-marathon marks in outdoors official events, between 1997 and 2020, for both sexes. The study included athletes who completed a race at least three consecutive times during the considered time range. After applying the eligibility criteria, the final sample comprised 24 half-marathon athletes (female: 12; male: 12).

### *Statistical analysis*

Descriptive information was expressed as mean (standard deviation), median (interquartile range), or frequency (%). Multivariate normality was tested and confirmed by the Doornik-Hansen, split by sex. Tracking was tested through two approaches: firstly, autocorrelations were performed, and cut-off points suggested by Malina was considered (*r* < ​0.3 ​= ​low correlation; 0.3 ​= *r* ​≤ ​0.60 ​= ​moderate correlation; *r* ​> ​0.60 ​= ​moderate -to- high correlation).^11^ Individual trajectories and fitted values were graphically presented. Pearson correlation values were converted from r to Fisher's *z*', mean values were calculated and the final value was transformed into r value. The cut of point used to determine the existence of stability was *r* ​> ​0.5. Statistical analysis was computed in STATA 16.0, and the significance level was set at 0.05. Secondly, Kappa Cohen was performed to verify performance stability along the three years, for both sexes. Three developmental canals (track) were considered: upper canal (> percentile 66); middle canal (> 33 percentile – 66 percentile); lower canal (< 33 percentile). Kappa results are analyzed based on Landis & Koch,^15^ being: *K* ​< ​0.40 ​= ​low; 0.40 ​< ​*K* ​< ​0.75 ​= ​medium; and *K* ​> ​0.75 ​= ​high. Statistical analysis was realized using Longitudinal Data Analysis software.

## Results

**Fig. 1:**
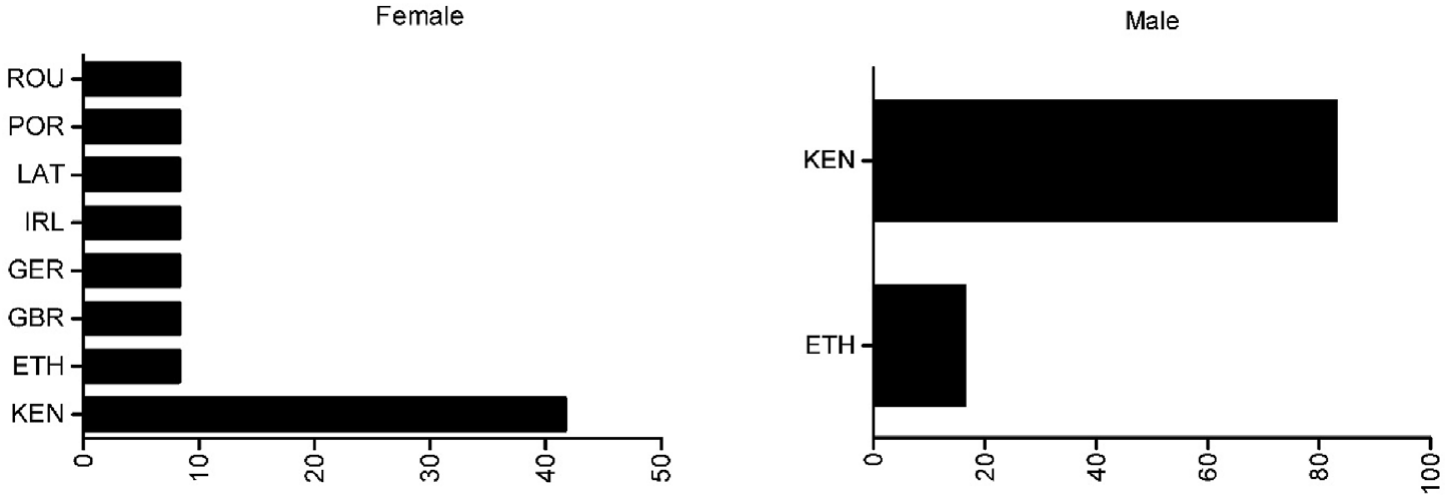
Athletes’ distribution by country.

**Fig. 2:**
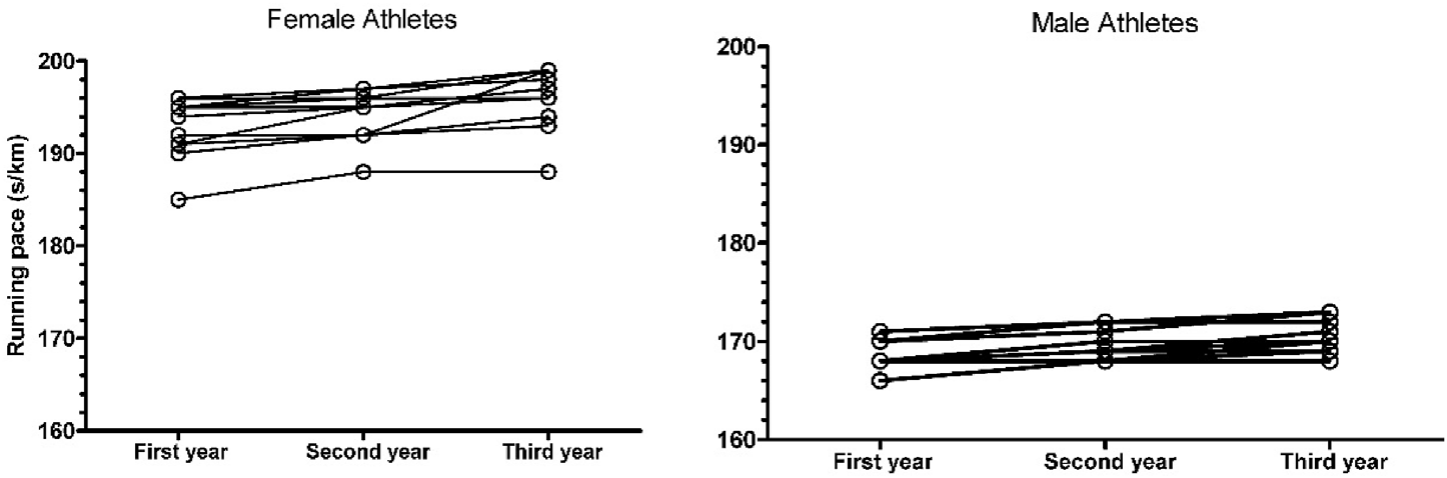
Individual trajectory of half-marathoner elite athletes during three years.

**Fig. 3:**
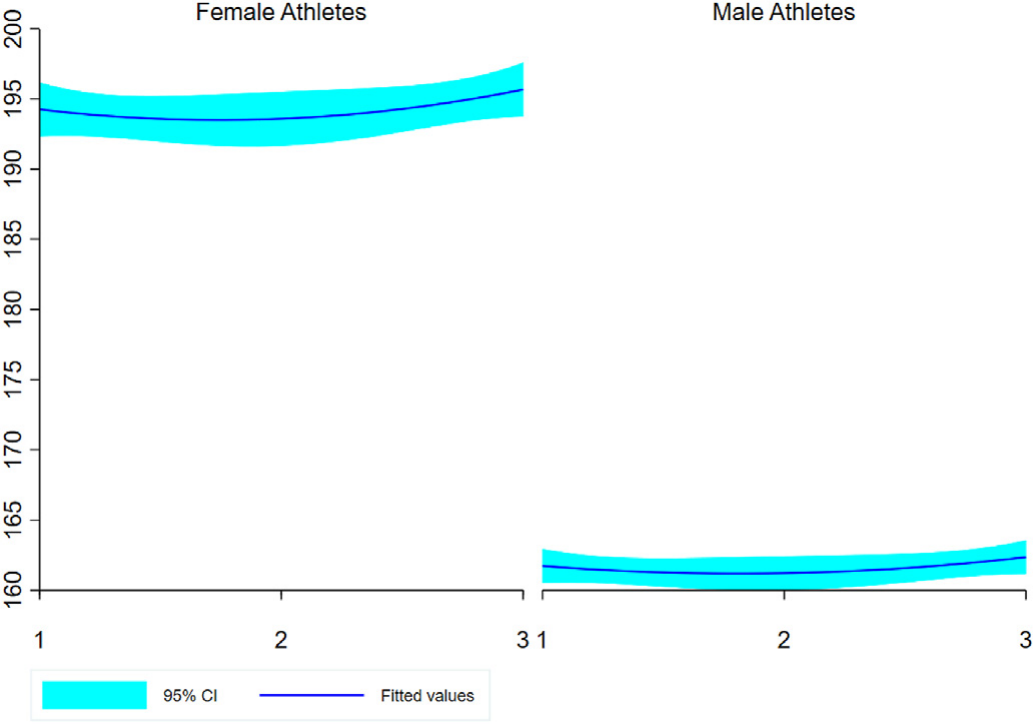
Fitted values to the trajectory in three years, with confidence interval.

Men were younger than women (24.2 ​± ​3.3 years and 28.1 ​± ​4.4 years, respectively), and for both sexes most of the athletes were from the African continent, especially from Kenya (women: 41.7%; men: 83.3%), followed by Ethiopia (women: 8.3%; men: 16.7%). Great Britain, Germany, Ireland, Latvia, Portugal, and Romania had one female athlete each (8.3%) (Fig. 1). Fig. 2 presents the individual trajectories for the three years. Female half-marathoners presented more variability during the years, with few athletes presenting an improvement or stability in performance over time. Among male half-marathoners, a high homogeneity was observed with similar trajectories; however, most of them presented a decrease in performance. Fig. 3 presents the fitted values, considering 95% of the confidence interval, for both sexes.

Performance stability results are presented for both sexes in Table 1. Based on Malina’s cut-off points, there was strong stability in performance (*r*’ woman ​= ​0.862; *r*’ men ​= ​0.867). In both sexes, a strong correlation was verified to the closest years (i.e., first and second year; second and third year).

**Table 1:** Autocorrelation results for both sexes, considering three years.

	Women			Men		
	*Pace 1*	*Pace 2*	*Pace 3*	*Pace 1*	*Pace 2*	*Pace 3*
*Pace 1*	1	0.935∗∗	0.790∗∗	1	0.871∗∗	0.867∗∗
Bias	0	−0.019	−0.079	0	−0.009	0.004
Std. Error	0	0.074	0.266	0	0.101	0.063
Lower	1	0.714	0.033	1	0.600	0.707
Upper	1	0.989	0.984	1	0.961	0.961
*Pace 2*	0.935∗∗	1	0.819∗∗	0.871∗∗	1	0.865∗∗
Bias	−0.019	0	−0.068	−0.009	0	−0.014
Std. Error	0.074	0	0.254	0.101	0	0.101
Lower	0.714	1	0.025	0.600	1	0.562
Upper	0.989	1	0.987	0.961	1	0.954
*Pace 3*	0.790∗∗	0.819∗∗	1	0.867∗∗	0.865∗∗	1
Bias	−0.079	−0.068	0	0.004	−0.014	0
Std. Error	0.266	0.254	0	0.063	0.101	0
Lower	0.033	0.025	1	0.707	0.562	1
Upper	0.984	0.987	1	0.961	0.954	1

∗∗ Correlation is significant at the 0.01 level; ∗ Correlation is significant at the 0.05 level; bootstrapping results were based in 5000 bootstrap samples; Pace 1, running pace in the first year; ​Pace 2, running pace in the second year; ​Pace 3, running pace in the third year.

Results for Kappa are presented for both sexes in Table 2. For women, the Kappa for each track indicated that athletes in the first track were more stable (*K* ​= ​0.37), compared to those from track three (*K* ​= ​0.12) and track two (*K* ​= ​0.03). For men, athletes in the first and second track were more stable (*K* ​= ​−0.333 and *K* ​= ​−0.204, for one and two, respectively). Overall weighted kappa results indicated lower stability in performance for both sexes (*K* ​= ​−0.191 and *K* ​= ​−0.245).

**Table 2:** Kappa Cohen results for each track, to both sexes.

Track	Women	Men
**>*P*66**	−0.125	−0.161
***P*33 – *P*66**	−0.037	−0.204
**<*P*33**	−0.371	−0.333
**Overall weighted *Kappa***	−0.191	−0.249
**95%*CI***	0.04–−0.42	0.005–−0.503

> *P*66 (percentile 66); *P*33 – *P*66 (percentile 33 and percentile 66); < *P*33 (below than percentile 33); 95%*CI* (95% of Confidence Interval).

## Discussion

The purpose of this study was to investigate the performance stability in elite half-marathoners of both sexes. We hypothesized that athletes of both sexes presented performance stability during the period considered. The main findings were that a decrease in correlation was observed in both groups over the years; a decrease in performance stability was shown during the three years assessed; and when athletes were stratified by performance level, based in percentile, those female and male athletes classified in the fastest group presented more stability in performance during the three years, however, in general, kappa analyses results showed lower stability in performance, for both sexes.

Age-related declines in runners was presented previously for master athletes^16^ non-professional athletes^17^ marathoners and high-elite athletes.^18^^,^^19^ Based on previous studies, it is important to take into account that most endurance athletes reach their peak performance in their 20s.^19^ For half-marathon, we have verified that peak performance occurred at the age of 25.6 ​± ​3.6 years for women and at 27.5 ​± ​4.4 years for men, respectively.^18^ In this sense, the low performance stability can be related to an increase in age and a decrease of this physiological index, once that a decrease in physiological characteristics that limit performance in endurance running (e.g., V̇O_2_max, maximum heart rate, stroke volume, and arteriovenous oxygen difference) is associated with increasing age.^16^

On the other hand, Bragada et al.^2^ reported a decrease in the performance of young athletes during two years follow-ups. A study conducted with young athletes in the UK showed a weak performance association between performance in U13 and U20 in all track and field disciplines.^20^ When performances of young Italian athletes were examined, Boccia et al.^21^ found that 0–5% of top-level (top 4%) senior long and high jumpers were considered top level when they were 12–13 years of age. These results were associated with the dynamic and non-linear characteristics of the performance.^22^ However, since few studies have been conducted to investigate tracking performance in endurance elite athletes, comparisons and explanations of results are limited.

The highest performance stability in athletes with the highest performance can be associated with a plethora of factors. The Pygmalion and Galatea effect was proposed in a socioecological area and refers, to a coach’s expectation in the athletes, and self-expectation in a domain, respectively.^23^^,^^24^ Despite that, most of the research published to explain performance in long-distance events is related to physiological indicators, the training maintenance in long-term training is also associated with psychological factors and contextual support. For example, research conducted by Durand-Bush^25^ with six Olympics and/or World Championships medalists show a number of factors that are associated with high-level performance maintenance: Training characteristics (i.e., technical, tactical, physical, and mental components and was influenced by quantity, quality, intensity, and recovery); contextual support (i.e., family, friends, parents, club and coaches, teammates and support staff); personal characteristics (i.e., self-confidence, motivation, creativity, and perseverance).

This study has some limitations. Firstly, the small sample size, since the reduced number of athletes who competed during three consecutive races, taking into account the time range considered, does not allow the generalization of the results. Second, given that we used secondary data, additional information regarding variables that could help in the explanation of the results were lacking, for example, differences related to training background, individual characteristics (nutritional habits, hydration during the race event), and context-related variables (i.e., temperature, humidity, windy, altimetry) are not available for the race events. On the other hand, to the best of the author’s knowledge, this is the first study conducted to investigate the tracking of performance in elite endurance athletes. In sports science, few studies were conducted with the purpose to investigate tracking in professional athletes. Information could be used to offer insights for researchers to develop new projects, considering the performance changes across the years. For coaches, the performance monitoring during the competition can be crossed with training characteristics and physiological index.

## Conclusion

These findings suggest that both female and male elite half-marathoners showed a low stability in performance during three consecutive events. When the data were stratified by performance level, the best ones presented the highest performance stability, i.e., they tend to maintain the same running pace across the years. Future studies need to consider longer periods (i.e., longer than three years), distance events (i.e., 5 ​km, 10 ​km, marathon), performance level (i.e., U18, U20), individual (physiological index changes) and training characteristics (i.e., load control, methods used) associated with athletes’ information.

## Submission statement

Our work submitted has not been published previously, is not under consideration for publication elsewhere, its publication is approved by all authors and tacitly or explicitly by the responsible authorities where the work was carried out, and, if accepted, it will not be published elsewhere including electronically in the same form, in English or in any other language, without the written consent of the copyright holder. All authors have read and agree with manuscript content.

## Authors’ contributions

MT conceptualized this study, conducted the literature search, perform the statistical analysis and wrote the original draft preparation. TNG conceptualized this study and reviewed and edited this paper. BK and PTN reviewed and edited this paper. All authors have read and approved the final version of the manuscript and agree with the order of presentation of the authors.

## Ethical approval

The institutional review board of St Gallen, Switzerland, approved this study (EKSG 01/06/2010). Since the study involved the analysis of publicly available data, the requirement for informed consent was waived.

## Conflict of interest

The author reports no conflicts of interest in this work.
